# Supplementary material for: Drug-Coated Balloon versus Plain Balloon Angioplasty in the Treatment of Infrainguinal Vein Bypass Stenosis: A Systematic Review and Meta-Analysis
Source: J Clin Med. 2022 Dec 22;12(1):87. doi: 10.3390/jcm12010087 (PMC9821647; doi:10.3390/jcm12010087)
Supplement: Supplementary file 1 [file jcm-12-00087-s001.zip › jcm-2071716-supplementary.pdf]

Search method　20220922

MEDLINE

("peripheral arterial disease"[MeSH Terms] OR ("arterial occlusive diseases"[MeSH Terms] AND 1963/01/01:2010/12/31[Date - Publication]) OR ("peripheral vascular diseases"[MeSH Terms] AND 1991/01/01:2010/12/31[Date - Publication]) OR ("femoral artery"[MeSH Terms] AND "popliteal artery"[MeSH Terms])) AND (("bypass"[All Fields] OR "bypassed"[All Fields] OR "bypasses"[All Fields] OR "bypassing"[All Fields]) AND ("graft s"[All Fields] OR "grafted"[All Fields] OR "graftings"[All Fields] OR "transplantation"[MeSH Subheading] OR "transplantation"[All Fields] OR "grafting"[All Fields] OR "transplantation"[MeSH Terms] OR "grafts"[All Fields] OR "transplants"[MeSH Terms] OR "transplants"[All Fields] OR "graft"[All Fields])) AND (("drug"[All Fields] AND ("coated"[All Fields] OR "coating"[All Fields] OR "coating s"[All Fields] OR "coatings"[All Fields]) AND ("balloon"[All Fields] OR "balloon s"[All Fields] OR "balloons"[All Fields])) OR ("drug"[All Fields] AND ("elutable"[All Fields] OR "elutant"[All Fields] OR "elute"[All Fields] OR "eluted"[All Fields] OR "elutent"[All Fields] OR "eluter"[All Fields] OR "eluters"[All Fields] OR "elutes"[All Fields] OR "eluting"[All Fields] OR "elution"[All Fields] OR "elutions"[All Fields]) AND ("balloon"[All Fields] OR "balloon s"[All Fields] OR "balloons"[All Fields])) OR (("paclitaxel"[MeSH Terms] OR "paclitaxel"[All Fields] OR "paclitaxel s"[All Fields] OR "paclitaxels"[All Fields]) AND ("coated"[All Fields] OR "coating"[All Fields] OR "coating s"[All Fields] OR "coatings"[All Fields]) AND ("balloon"[All Fields] OR "balloon s"[All Fields] OR "balloons"[All Fields]))) AND "angioplasty, balloon"[MeSH Terms]

Translations

bypass: "bypass"[All Fields] OR "bypassed"[All Fields] OR "bypasses"[All Fields] OR "bypassing"[All Fields]

graft: "graft's"[All Fields] OR "grafted"[All Fields] OR "graftings"[All Fields] OR "transplantation"[Subheading] OR "transplantation"[All Fields] OR "grafting"[All Fields] OR "transplantation"[MeSH Terms] OR "grafts"[All Fields] OR "transplants"[MeSH Terms] OR "transplants"[All Fields] OR "graft"[All Fields]

coated: "coated"[All Fields] OR "coating"[All Fields] OR "coating's"[All Fields] OR "coatings"[All Fields]

balloon: "balloon"[All Fields] OR "balloon's"[All Fields] OR "balloons"[All Fields]

eluting: "elutable"[All Fields] OR "elutant"[All Fields] OR "elute"[All Fields] OR "eluted"[All Fields] OR "elutent"[All Fields] OR "eluter"[All Fields] OR "eluters"[All Fields] OR "elutes"[All Fields] OR "eluting"[All Fields] OR "elution"[All Fields] OR "elutions"[All

Fields]
balloon: "balloon"[All Fields] OR "balloon's"[All Fields] OR "balloons"[All Fields]
paclitaxel: "paclitaxel"[MeSH Terms] OR "paclitaxel"[All Fields] OR "paclitaxel's"[All Fields] OR "paclitaxels"[All Fields]
coated: "coated"[All Fields] OR "coating"[All Fields] OR "coating's"[All Fields] OR "coatings"[All Fields]
balloon: "balloon"[All Fields] OR "balloon's"[All Fields] OR "balloons"[All Fields]
Angioplasty, Balloon[MH]: "angioplasty, balloon"[MeSH Terms]

Cochrane Library 20220922

	Search	Hits
#1	(peripheral arterial disease)	
#2	(femoropopliteal artery disease)	
#3	(infrainguinal artery disease)	
#4	bypass graft	
#5	(#1 or #2 or #3) and #4	
#6	(drug coated balloon)	
#7	(drug eluting balloon)	
#8	(paclitaxel coated balloon)	
#9	#6 or #7 or #8	
#10	#5 and #9 in Cochrane Reviews, Trials	
